# Differential Gene Expression of Three Mastitis-Causing *Escherichia coli* Strains Grown under Planktonic, Swimming, and Swarming Culture Conditions

**DOI:** 10.1128/mSystems.00064-16

**Published:** 2016-08-09

**Authors:** John D. Lippolis, Brian W. Brunelle, Timothy A. Reinhardt, Randy E. Sacco, Tyler C. Thacker, Torey P. Looft, Thomas A. Casey

**Affiliations:** a Ruminant Diseases and Immunology Research Unit, National Animal Disease Center, Agricultural Research Service (ARS), United States Department of Agriculture (USDA), Ames, Iowa, USA; b Food Safety and Enteric Pathogens Research Unit, National Animal Disease Center, Agricultural Research Service (ARS), United States Department of Agriculture (USDA), Ames, Iowa, USA; c Bacterial Diseases of Livestock Research Unit, National Animal Disease Center, Agricultural Research Service (ARS), United States Department of Agriculture (USDA), Ames, Iowa, USA; Argonne National Laboratory

**Keywords:** motility, pathogenesis, virulence

## Abstract

Bacterial motility is thought to play an important role in virulence. We have previously shown that proficient bacterial swimming and swarming *in vitro* is correlated with the persistent intramammary infection phenotype observed in cattle. However, little is known about the gene regulation differences important for different motility phenotypes in *Escherichia coli*. In this work, three *E. coli* strains that cause persistent bovine mastitis infections were grown in three media that promote different types of motility (planktonic, swimming, and swarming). Using whole-transcriptome RNA sequencing, we identified a total of 935 genes (~21% of the total genome) that were differentially expressed in comparisons of the various motility-promoting conditions. We found that approximately 7% of the differentially expressed genes were associated with iron regulation. We show that motility assays using iron or iron chelators confirmed the importance of iron regulation to the observed motility phenotypes. Because of the observation that *E. coli* strains that cause persistent infections are more motile, we contend that better understanding of the genes that are differentially expressed due to the type of motility will yield important information about how bacteria can become established within a host. Elucidating the mechanisms that regulate bacterial motility may provide new approaches in the development of intervention strategies as well as facilitate the discovery of novel diagnostics and therapeutics.

**IMPORTANCE** Bacteria can exhibit various types of motility. It is known that different types of motilities can be associated with virulence. In this work, we compare gene expression levels in bacteria that were grown under conditions that promoted three different types of *E. coli* motility. Better understanding of the mechanisms of how bacteria can cause an infection is an important first step to better diagnostics and therapeutics.

## Abstract (precis)

Bacteria can exhibit various types of motility. It is known that different types of motilities can be associated with virulence. In this work, we compare gene expression levels in bacteria that were grown under conditions that promoted three different types of *E. coli* motility. Better understanding of the mechanisms of how bacteria can cause an infection is an important first step to better diagnostics and therapeutics.

## INTRODUCTION

The manners in which bacteria can move are diverse (1, 2). Two of the best-described types of bacterial motility are swimming and swarming. Swimming motility is defined as that exhibited by individual bacteria propelled by rotating flagella in liquid or semisolid media. Swimming bacteria use chemotaxis to find nutrients and avoid toxic environments. Swarming represents the coordinated motility of a dense group of bacteria (2, 3) and, like swimming, is mediated by flagella. However, chemotaxis is suppressed during swarming. Interestingly, swarming bacteria can demonstrate increased resistance to antibiotics (3). The type of bacterial motility is thought to correlate with the induction of various virulence determinants, chemotaxis signaling pathways, and surfactant synthesis and the acquisition of required nutrients such as iron (4).

Mammary-pathogenic *Escherichia coli* (MPEC) is a leading cause of acute mastitis in dairy animals (5). Coliform mastitis is considered to occur in an ascending manner, meaning that motility is thought to contribute to virulence by enabling MPEC to disseminate from the site of infection (teat canal) into the milk ducts and alveolar system of the mammary gland. Mastitis caused by *E. coli* is typically transient in duration, but persistent intramammary infections can occur (6). Strains of MPEC that cause persistent intramammary infections have been shown to invade cultured mammary epithelial cells more effectively than strains that cause transient infections (7). In addition, MPEC strains that cause persistent infections have been shown to have greater motility *in vitro* as demonstrated by increased rates of swimming and swarming compared to MPEC strains that cause transient mastitis and displayed little to no motility (8). Other pathogenic bacteria that exhibit greater motility in various assays are thought to have enhanced virulence compared to less-motile strains (4, 9). Therefore, understanding motility is an important part of understanding bacterial virulence.

In this work, we studied the changes in gene expression in MPEC strains isolated from media that promote different bacterial motility. The goal of this study was to determine the gene expression changes in highly motile MPEC strains under planktonic, swimming, and swarming growth conditions. Based on the finding of a large number of differentially expressed genes associated with the ferric uptake regulator (Fur), we explored the effects of iron on motility. Since motility has been long associated with virulence, we anticipate that this increased understanding of motility will lead to greater insight into the processes of pathogenicity of mammary-pathogenic *E. coli* (4, 9).

## RESULTS

**FIG 1:**
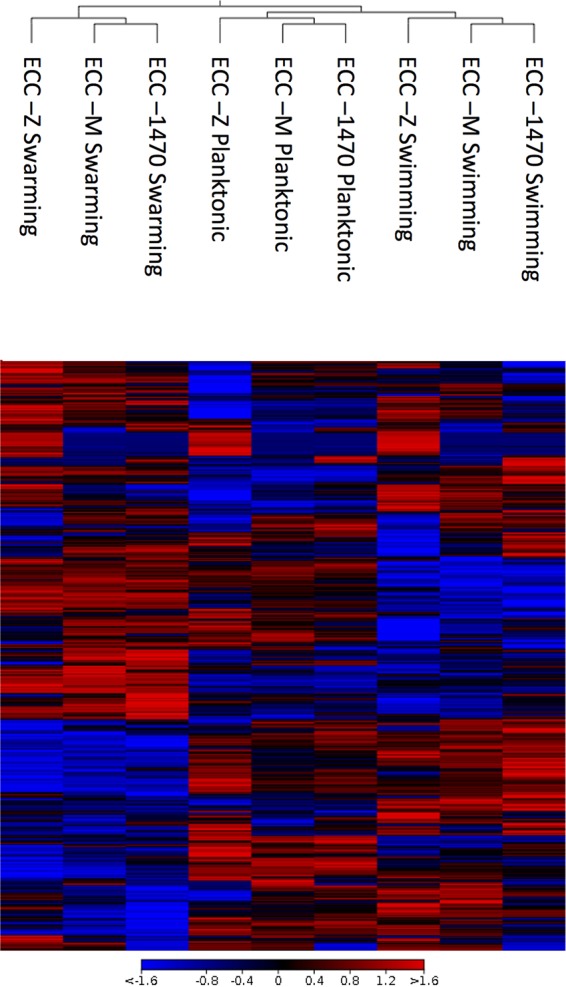
Heat map of gene expression. Comparisons of gene expression patterns of the whole transcriptome of the 3 MPEC strains in the 3 motility groups are shown. The gradient is representative of the expression differences between the samples. Gene expression clusters are categorized by motility, as denoted by the separation of the groups in the tree (see top of figure).

Three MPEC strains were grown separately in liquid media (Luria-Bertani [LB]), on semisolid agar plates that promote the swimming phenotype, and on semisolid agar plates that promote the swarming phenotype. Bacteria were harvested from each of the growth media, and RNA was isolated and sequenced. Sequence read information was mapped to 4,435 genes in *E. coli* reference strain MG1655 and plasmid CP009167, a plasmid from previous described mastitis-causing *E. coli* strain 727. A heat map was generated through hierarchical clustering of gene expression pattern similarities for the three MPEC strains under each of the three motility conditions for all the genes identified in the study (Fig. 1). Clustering by similar gene expression profiles showed that the samples all grouped by motility phenotype.

**FIG 2:**
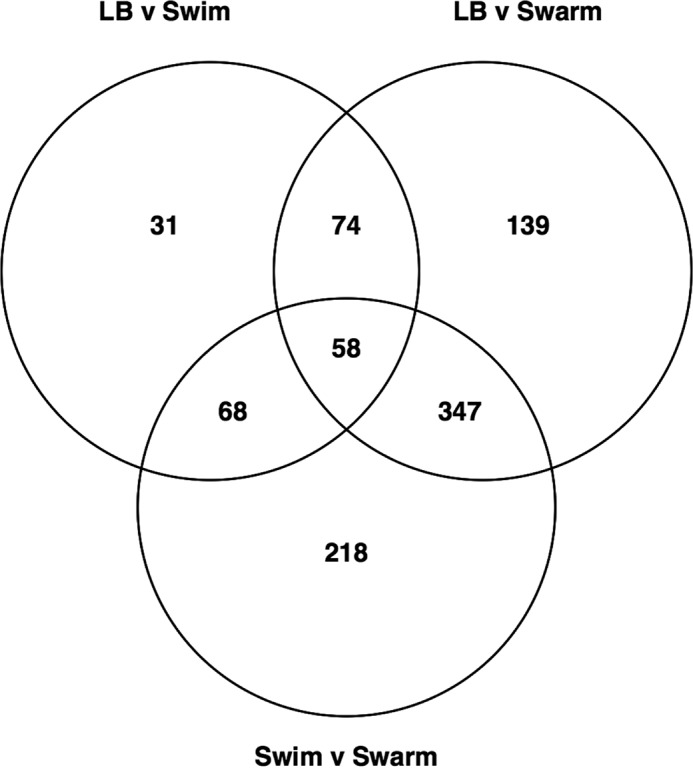
Venn diagram of differentially expressed genes. The Venn diagram shows the 935 genes differentially expressed (FDR *P* ≤ 0.05) among our three comparison groups (LB versus swim, LB versus swarm, and swim versus swarm).

The three MPEC strains were used as the biological replicates under each growth condition to determine significant gene expression changes between the three motility phenotypes. We found 935 genes that showed significant expression differences (false-discovery rate [FDR], *P* ≤ 0.05) in comparisons of gene expression levels of any two of the three growth conditions (see Table S1 in the supplemental material). Figure 2 is a Venn diagram that indicates the numbers of genes with significant expression changes associated with growth under the three different motility conditions. Similarly, Table 1 shows the number of significant differentially expressed genes divided by numbers corresponding to the comparison groups (LB versus swim, LB versus swarm, or swim versus swarm) and the direction of the expression change. Of the 935 total differentially expressed genes, 231 genes were found in the comparison of bacterial motility in liquid LB versus swimming, 618 in the comparison of LB versus swarming, and 691 in the comparison of swimming versus swarming (Table 1).

10.1128/mSystems.00064-16.1 Table S1  Complete list of all genes aligned with RNA-Seq data. Gene identifier (ID), genome (*E. coli* strain MG1655 or plasmid CP009167), and start (chromosome start location) data are presented under the first three column headings. Those are followed by the data for the three comparison groups in two columns each corresponding to FC (fold change) and FDR (false-discovery rate) as indicated. The data under the column headings Type, Function, and Description are from an EcoCyc database. Download Table S1, XLSX file, 0.7 MB. Copyright © 2016 Lippolis et al. 2016 Lippolis et al. This content is distributed under the terms of the Creative Commons Attribution 4.0 International license.

**TABLE 1:**
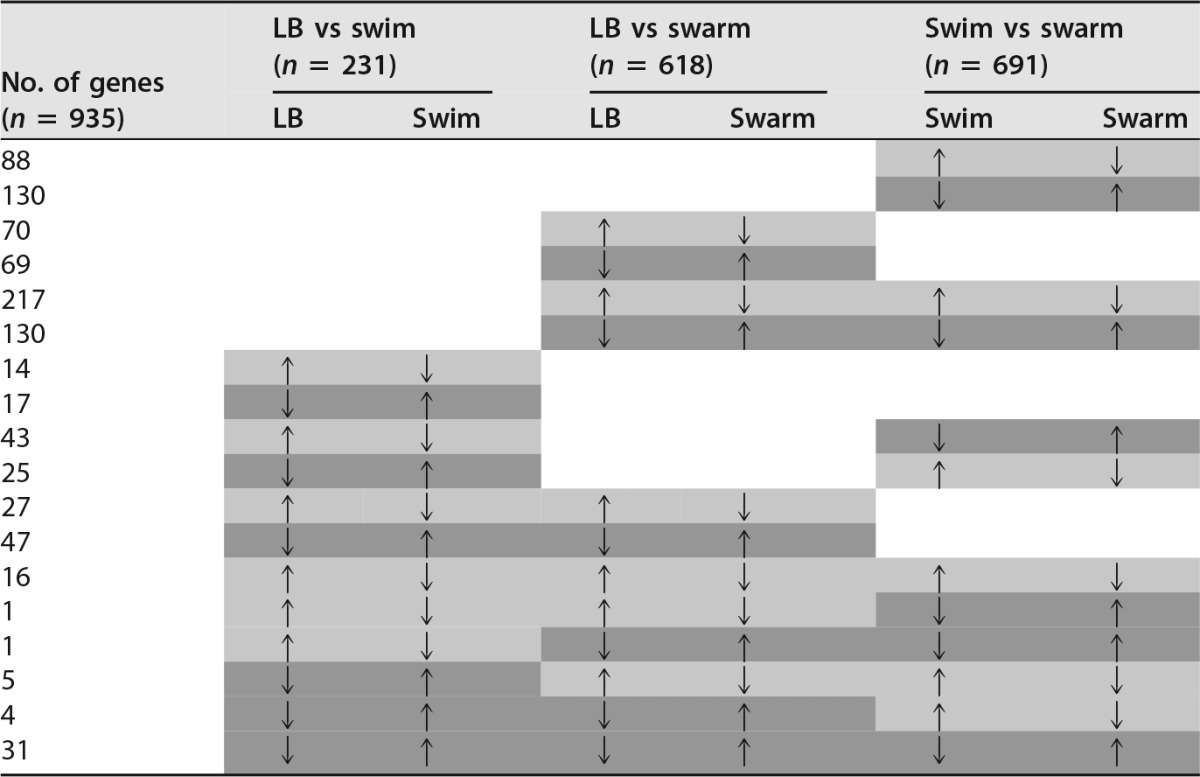
Number of differentially expressed genes and their direction of regulation for LB versus swim, LB versus swarm, and swim versus swarm^a^

a Light gray boxes indicate upregulation for the first parameter in the comparison and downregulation for the second parameter; dark gray boxes indicate downregulation for the first parameter in the comparison and upregulation for the second parameter.

We analyzed our data set using David Bioinformatics Resource to compare functional groups identified using enriched Gene Ontology (GO) terms (10, 11). The positive and negative expression changes from each of the three comparison groups were analyzed independently and are summarized in Tables 2 and 3. Table 2 shows only the highly significant (FDR *P* ≤ 0.001) molecular function GO terms. The majority of enriched molecular function GO terms found in Table 2 are related to ion binding, particularly that of iron. Table 3 shows the top 20 biological process GO terms that were differentially expressed. Included in the list of enriched biological process GO terms are anaerobic and aerobic respiration, carbohydrate catabolic process, tricarboxylic acid (TCA) cycle, and iron transport.

**TABLE 2:** Gene expression changes categorized by molecular-function GO terms^a^

GO term	Expression change in comparison of:											
	LB versus swim				LB versus swarm				Swim versus swarm			
	Negative		Positive		Negative		Positive		Negative		Positive	
	Count	FDR	Count	FDR	Count	FDR	Count	FDR	Count	FDR	Count	FDR
Iron ion binding			23	7.19E-06	37	7.32E-08	59	1.02E-22	35	1.98E-05	52	1.71E-14
Metal ion binding					67	4.66E-06	85	1.70E-12	67	4.34E-04	101	6.31E-17
Cation binding					70	8.70E-07	85	1.02E-11	71	4.85E-05	101	5.24E-16
Ion binding					70	1.09E-06	85	1.39E-11	71	5.99E-05	101	7.60E-16
Transition metal ion binding					56	2.88E-06	74	6.29E-14	54	9.03E-04	81	2.54E-14
Ligase activity, forming carbon-nitrogen bonds							13	1.95E-04			16	1.40E-06
4 Iron, 4 sulfur cluster binding					25	4.70E-06	21	6.93E-03	24	1.63E-04		
Nucleotide binding											82	6.20E-05
Cofactor binding											48	1.09E-04
Iron-sulfur cluster binding					29	1.10E-04	26	2.05E-02	27	9.94E-03		
Metal cluster binding					29	1.10E-04	26	2.05E-02	27	9.94E-03		
2,3-Bisphosphoglycerate-dependent phosphoglycerate mutase activity							5	1.68E-04			5	2.92E-04
Magnesium ion binding											27	1.90E-04
Inorganic cation transmembrane transporter activity							17	4.93E-04				
Calcium ion binding									8	9.42E-04		
Carbohydrate-importing ATPase activity									8	1.85E-03		
Metal ion transmembrane transporter activity							14	1.95E-03			14	8.38E-03
Carbohydrate-transporting ATPase activity									8	3.41E-03		

a Negative, higher transcript amount under the first growth condition; positive, higher transcript amount under the second growth condition. Count data represent the number of genes from each GO term. All categories showed significant enrichment (FDR *P*, ≤0.001).

**TABLE 3:** Gene expression changes categorized by biological-process GO terms^a^

GO term	Expression change in comparison of:											
	LB versus swim				LB versus swarm				Swim versus swarm			
	Negative		Positive		Negative		Positive		Negative		Positive	
	Count	FDR	Count	FDR	Count	FDR	Count	FDR	Count	FDR	Count	FDR
Carbohydrate catabolic process			15	4.15E-02	55	9.27E-27			69	2.88E-38		
Nitrogen compound biosynthetic process							74	2.20E-25			72	2.65E-20
Amine biosynthetic process			15	2.79E-02			48	6.76E-19			41	2.76E-11
Anaerobic respiration	8	2.63E-03			27	2.24E-18			25	6.47E-15	18	1.15E-06
Iron ion transport			10	2.63E-04			26	1.27E-17			26	2.62E-16
Di- and trivalent inorganic cation transport			10	1.08E-03			26	8.61E-16			26	1.69E-14
Carboxylic acid biosynthetic process							47	9.50E-16			48	2.70E-14
Organic acid biosynthetic process							47	1.13E-15			48	3.20E-14
Aerobic respiration			16	5.86E-12			24	1.71E-15			19	7.55E-09
Cellular amino acid biosynthetic process							41	1.57E-14			40	8.12E-12
Transition metal ion transport			10	7.47E-03			27	2.38E-14			28	4.93E-14
Energy derivation by oxidation of organic compounds	10	2.96E-02	19	1.28E-07	34	6.18E-14	26	2.16E-06	34	1.58E-12	28	1.33E-06
Peptidyl-aspartic acid modification							10	3.22E-13				
Peptidyl-cysteine modification							10	3.22E-13				
Peptidyl-l-beta-methylthioaspartic acid biosynthetic process from peptidyl-aspartic acid							10	3.22E-13				
Generation of precursor metabolites and energy			21	3.86E-05	42	2.82E-12	37	1.77E-07	38	4.25E-08	40	1.12E-07
Cellular respiration			18	1.72E-07	30	8.45E-12	26	1.78E-07	29	8.17E-10	26	2.42E-06
Sulfur metabolic process							26	1.06E-10			28	3.20E-11
Metal ion transport							32	1.25E-09			31	1.58E-07
Tricarboxylic acid cycle			9	7.52E-05			15	2.14E-08				

a Negative, higher transcript amount under the first growth condition; positive, higher transcript amount under the second growth condition. Count data represent the number of genes from each GO term. All categories showed significant enrichment (FDR *P*, ≤0.001).

Due to the importance of iron regulation in pathogenic bacteria and the prevalence of differentially expressed iron binding and transport genes in our data set, we compared our data to a data set of genome-wide ferric uptake regulator (Fur) binding sites. In work reported by Seo and coworkers, 81 genes were identified as being a part of the Fur transcriptional regulatory network (12); we found 80 of those genes present in our data set (see Table S2 in the supplemental material). Of the 80 genes from the Fur regulatory network for which we have gene expression data, 63 showed significant expression change, and all of those changes were associated with swarming (either LB versus swarm or swim versus swarm). In the LB-versus-swimming comparison, 22 Fur-associated genes were differentially expressed.

10.1128/mSystems.00064-16.2 Table S2  List of genes that have been associated with the Fur regulon (12). Gene ID, genome (*E. coli* strain MG1655), and start (chromosome start location) data are presented under the first three column headings. Those are followed by the data for the three comparison groups in two columns each corresponding to FC (fold change) and FDR (false-discovery rate) as indicated. Data with an FDR *P* of ≤0.05 are highlighted. Negative, higher transcript amount under the first growth condition; positive, higher transcript amount under the second growth condition. Download Table S2, XLSX file, 0.1 MB. Copyright © 2016 Lippolis et al. 2016 Lippolis et al. This content is distributed under the terms of the Creative Commons Attribution 4.0 International license.

In Table S3, we compared our data with the data set of Inoue and coauthors (13). They identified genes important for swimming and/or swarming using a comprehensive collection of gene-disrupted *E. coli* K-12 mutants. Our data set contained 677 genes that were also contained in the Inoue data set; 138 of those genes were differentially expressed in one of our comparison groups. Inoue and coworkers divided their gene list into three groups: disrupted nonessential genes (*n* = 510) that led to a moderate repression of swarming with no effect on swimming, those nonessential genes (*n* = 216) that led to a strong repression of swarming with no effect on swimming, and those nonessential genes (*n* = 78) that led to a strong repression of both swimming and swarming (13). In our gene expression data, we found 85, 51, and 2 genes that matched those categories, respectively.

10.1128/mSystems.00064-16.3 Table S3  List of genes that have been associated with the Inoue data set (13). Gene ID, genome (*E. coli* strain MG1655 or plasmid CP009167), and start (chromosome start location) data are presented under the first three column headings. Those are followed by the data for the three comparison groups in two columns each corresponding to FC (fold change) and FDR (false-discovery rate) as indicated. Data with an FDR *P* of ≤0.05 are highlighted. The classifications used in the Inoue paper are indicated in the next column. There are three classifications: moderately repressed swarming, strongly repressed swarming and swimming, and strongly repressed swarming and no-effect swimming. Negative, higher transcript amount under the first growth condition; positive, higher transcript amount under the second growth condition. Download Table S3, XLSX file, 0.1 MB. Copyright © 2016 Lippolis et al. 2016 Lippolis et al. This content is distributed under the terms of the Creative Commons Attribution 4.0 International license.

**FIG 3:**
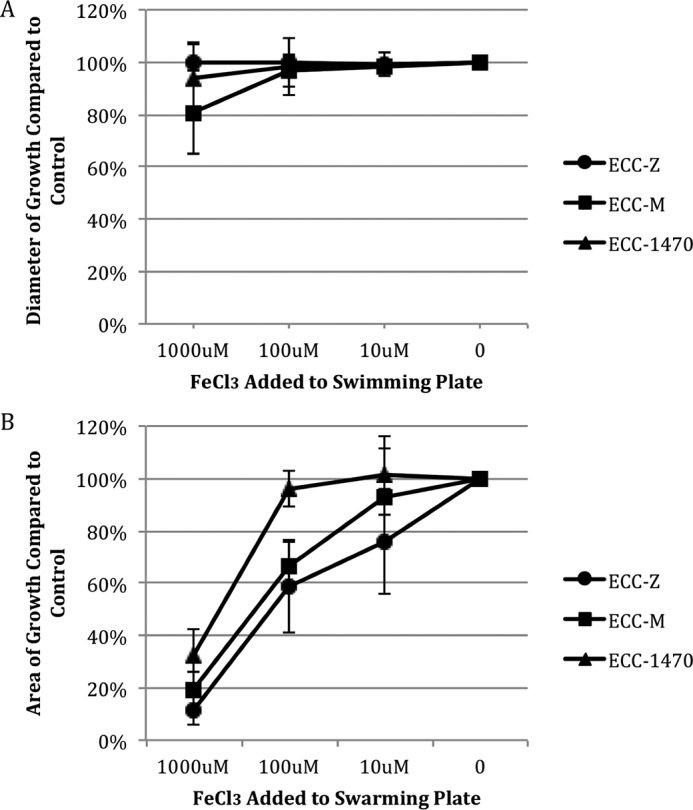
Effect of FeCl_3_ treatment on swimming and swarming of three MPEC strains. MPEC strains ECC-Z, ECC-M, and ECC-1470 were grown on swimming (A) and swarming (B) plates with 1,000 µM, 100 µM, 10 µM, or no added FeCl_3_. There were no significant FeCl_3_ treatment effects seen for any of the bacterial strains on the swimming plates shown in panel A. All three bacterial strains showed a significant (*P* ≤ 0.05) FeCl_3_ treatment effect on the swarming plates shown in panel B.

**FIG 4:**
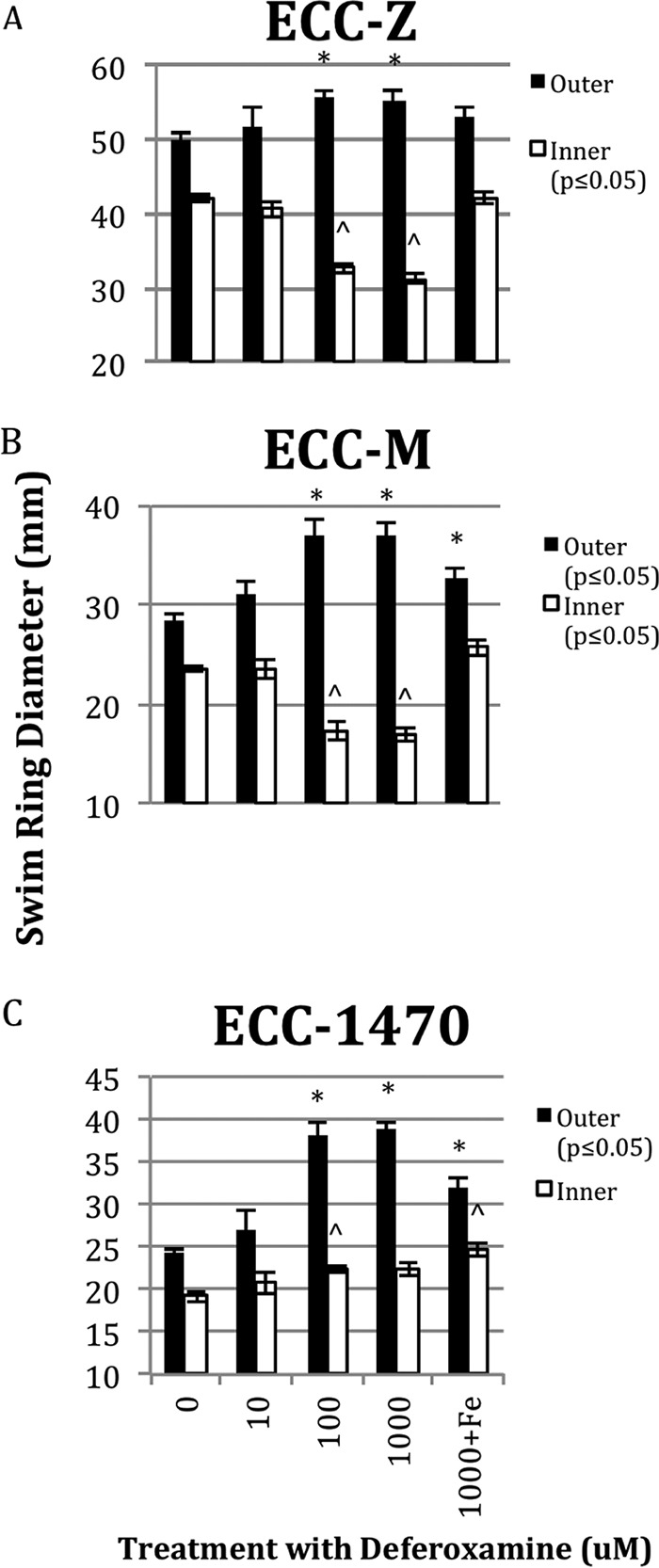
Effect of deferoxamine on swimming of three MPEC strains. MPEC strains ECC-Z (A), ECC-M (B), and ECC-1470 (C) were grown on swimming plates with 1,000 µM, 100 µM, or 10 µM deferoxamine, no added deferoxamine, or 1,000 µM deferoxamine plus 1,000 µM FeCl_3_. Significant treatment effects are indicated in the legend. Significant differences between the control (0) and other treatments are indicated with an asterisk (*) for the outer rings and a caret (^) for the inner ring.

**FIG 5:**
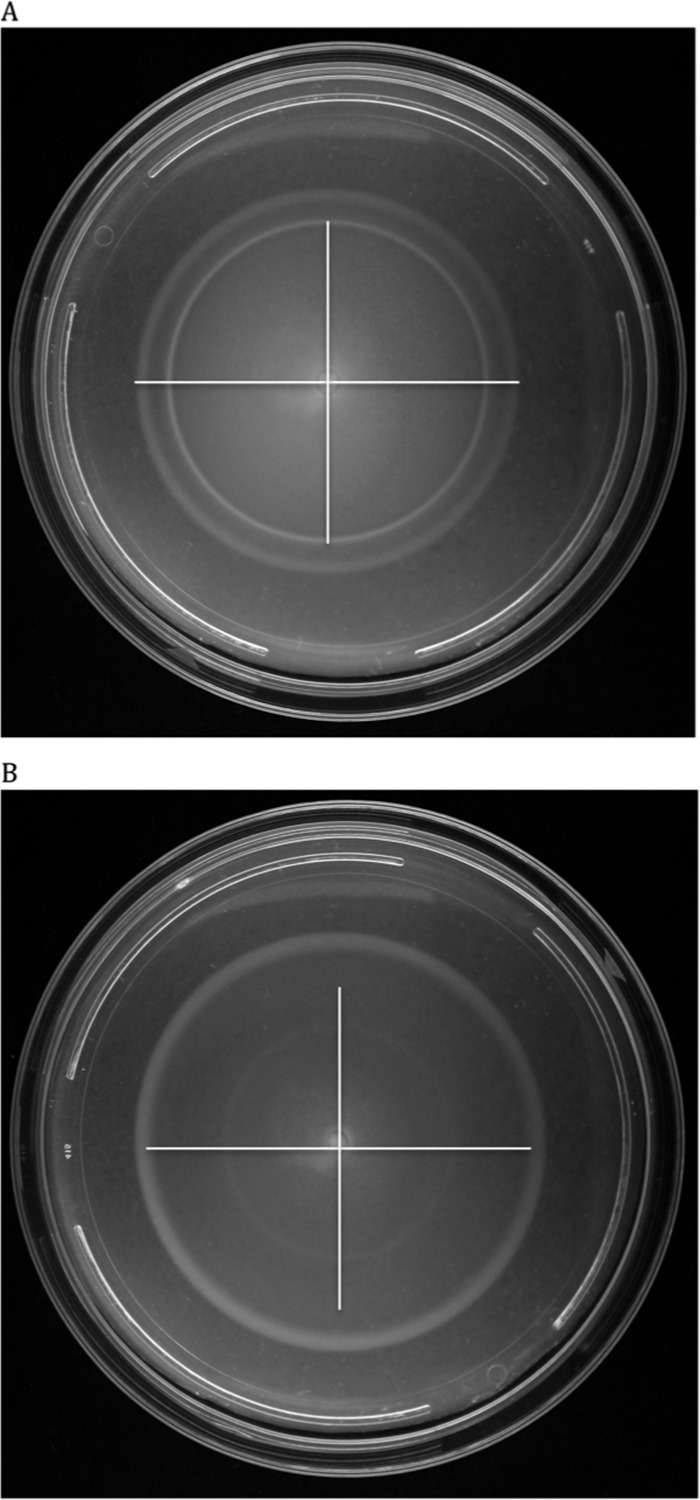
Differences in swimming motility due to iron chelation. Representative data are shown for strain ECC-Z grown on normal swim media (A) and on swim media with 1,000 µM deferoxamine (B). The horizontal and vertical lines in panel A show the sizes of the inner and outer rings. These same lines were copied into panel B to illustrate the size changes of both rings in the treated plate.

**FIG 6:**
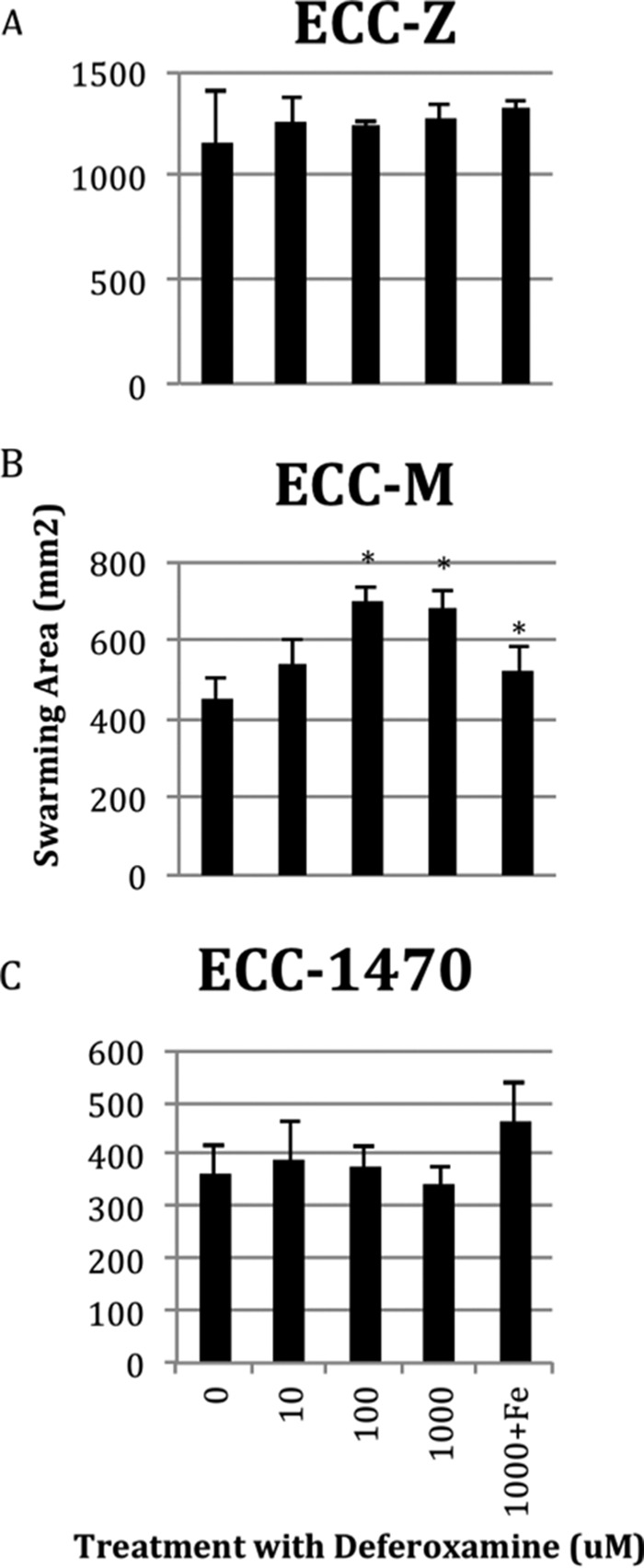
Effect of deferoxamine on swarming of three MPEC strains. MPEC strains ECC-Z (A), ECC-M (B), and ECC-1470 (C) were grown on swarming plates with 1,000 µM, 100 µM, or 10 µM deferoxamine, no added deferoxamine, or 1,000 µM deferoxamine plus 1,000 µM FeCl_3_. A treatment effect was significant (*P* ≤ 0.05) only for ECC-M (B). Significant differences between the control (0) and other treatments are indicated with an asterisk (*).

Given the number of iron-related genes that showed significant expression changes (63 genes), we wanted to better understand the effect of iron on motility. To do so, we added iron in the form of FeCl_3_ to swimming and swarming plates at concentrations of 0, 10, 100, and 1,000 µM and measured changes in the motility phenotype for the three MPEC strains. There was no significant change in the swimming results for any of the bacterial strains at the highest concentration of added FeCl_3_ of 1,000 µM (Fig. 3A). In contrast, all three MPEC strains had significantly reduced swarming areas at the 1,000 µM FeCl_3_ concentration and ECC-M was significantly inhibited at a FeCl_3_ concentration of 100 µM (Fig. 3B). Figures 4 and 5 show the effect of adding deferoxamine (iron chelator) at concentrations of 0, 10, 100, and 1,000 µM to swimming and swarming plates, respectively. Bacterial swimming plates are known to have multiple concentric circles of growth. We measured the diameters of the outer and inner (second) rings on plates that contained various concentrations of deferoxamine. Generally, when these three MPEC strains were grown on swimming plates, increasing concentrations of deferoxamine resulted in a larger outer ring and a smaller inner ring. Representative data are shown in Fig. 5. The outer rings showed a significant treatment effect of deferoxamine (*P* ≤ 0.05) for MPEC strains ECC-M and ECC-1470. The outer ring for strain ECC-Z did not show a significant treatment effect (*P* = 0.09). The inner ring showed a significant deferoxamine treatment effect for strains ECC-Z and ECC-M, whereas there was no treatment effect seen with ECC-1470. Figure 6 shows the effect of deferoxamine on the swarming of our three MPEC strains. Strains ECC-Z and ECC-1470 exhibited no change in swarming with the addition of deferoxamine. In contrast, strain ECC-M showed a significant increase in the swarming area.

Using the data from EcoCyc, we identified a data set of 43 small regulatory RNAs or antisense RNAs (14). These RNAs are thought to modulate gene expression. Table S4 in the supplemental material contains expression data for these RNAs. Eight of these genes had a significant expression difference in at least one of our comparison groups. Of particular interest was the upregulation of *ryhB* in the LB-versus-swarm comparison due to this gene’s function in mediating positive Fur regulon responses. Data corresponding to additional potential antisense transcripts are included in Table S5. There were 183 genes in the antisense orientation that showed a significant expression difference in our comparison groups.

10.1128/mSystems.00064-16.4 Table S4  List of genes that have been associated with the small regulatory RNA or known antisense RNA. Gene ID, genome (*E. coli* strain MG1655), and start (chromosome start location) data are presented under the first three column headings. Those are followed by the data for the three comparison groups in two columns each corresponding to FC (fold change) and FDR (false-discovery rate) as indicated. Data with an FDR *P* of ≤0.05 are highlighted. Description data are from the EcoCyc database. Small regulatory RNAs were identified by searching the EcoCyc database for the term “small regulatory RNA” and by searching our database for the term “antisense” and manually curating the data. Download Table S4, XLSX file, 0.01 MB. Copyright © 2016 Lippolis et al. 2016 Lippolis et al. This content is distributed under the terms of the Creative Commons Attribution 4.0 International license.

10.1128/mSystems.00064-16.5 Table S5  List of both sense and antisense matches to genes. Gene ID, genome (*E. coli* strain MG1655), and start (chromosome start location) data are presented under the first column headings. Those are followed by the data for the three comparison groups presented in four columns. FC indicates fold change, FDR indicates false-discovery rate, and A- indicates antisense data. Starting in column P is the read information for the three bacterial strains ECC-Z (1), ECC-M (2), and ECC-1470 (3) grown in LB or on swimming or swarming plates. All antisense data are highlighted in gray. Download Table S5, XLSX file, 0.1 MB. Copyright © 2016 Lippolis et al. 2016 Lippolis et al. This content is distributed under the terms of the Creative Commons Attribution 4.0 International license.

## DISCUSSION

Bacteria can employ several different mechanisms for motility, such as swimming, swarming, twitching, gliding, and sliding, that may be important for host colonization (2). Swimming is an individual motility behavior and is seen in liquid or plates with a very low (0.3%) agar concentration. Swarming is a group motility behavior where bacteria move in side-by-side groups called rafts (2). Swarming bacteria move along the surface of the agar dish, whereas swimming bacteria move within the agar. Both swimming and swarming motilities are driven by flagella. The ability to swim or swarm is thought to be associated with increased pathogenicity (4). Swarming bacteria have also exhibited resistance to multiple antibiotics (15). Our previous work demonstrated that strains of mastitis-causing *E. coli* showed different swimming and swarming abilities that correlated with persistent versus transient infections; interestingly, the transient-infection isolates displayed little to no ability to swim or swarm (8). In this work, we explore the gene expression changes due to growth on different motility-promoting media in three MPEC strains that cause persistent mammary gland infections.

As demonstrated in Table 2, iron utilization genes comprised one of the most affected groups of differentially regulated genes in comparisons of MPEC strains performed under planktonic, swimming, and swarming growth conditions. Previous work reported that upregulation of iron acquisition genes was observed when *Salmonella enterica* serovar Typhimurium was grown under swarming conditions compared to nonswarming conditions (16). The Fur regulator is thought to be a key element that regulates nutritional and virulence factors to control the bacterium’s adaptation to various environments, especially those inside a host (17). In this work, we have determined expression data on 80 of the 81 genes shown to be part of the Fur regulon (see Table S2 in the supplemental material), and 63 (79%) of these genes were differentially regulated in comparisons of swarming to the other motility growth conditions. We demonstrate that approximately 7% of all differentially regulated genes observed in this study are associated with the iron regulatory system. We also show that 22 Fur-regulated genes were differentially expressed in comparisons of planktonic-growth conditions with swimming conditions. All of the genes differentially regulated in the LB-versus-swim group were also differentially regulated in the LB-versus-swarm group. Most were expressed significantly (FDR *P* ≤ 0.05) more highly under swarming conditions than under swimming conditions. A few genes were upregulated equally under swimming and swarming conditions, and among those were three genes (*ariR*, *ycgZ*, and *ymgA*) that encode connector proteins for RcsB regulation of biofilm and acid resistance (18, 19). Based on the Fur regulon gene expression data, it would appear that swimming is an intermediate motility type between planktonic and swarming motilities.

RyhB is a small antisense RNA that, together with Fur, regulates a set of target genes that have been shown to play an important role in pathogenesis (17). Under iron-rich conditions, Fur acts as a negative regulator of *ryhB*. Table S4 in the supplemental material contains a list of 43 small antisense or other identified regulatory antisense RNAs, 8 of which were significantly differentially expressed under at least one motility condition. The functions of some of these small regulatory RNAs include regulation of the *rpoS* global regulator, activation of genes that detoxify oxidative damage, and regulation of the toxic proteins encoded by *lbsAB*. There is currently no data available regarding the potential role of small antisense RNAs in mastitis. Additionally, we have made available our complete antisense data set (Table S5).

To further understand the role of iron in swimming and swarming, we grew our three MPEC strains on plates with added iron (FeCl_3_) or with an iron chelator. The iron chelator we used was deferoxamine, a bacterial siderophore produced by *Streptomyces pilosus* that also has a medical application as a chelating agent to treat acute iron poisoning. Addition of FeCl_3_ to swimming plates resulted in no significant effect on the ability of any of the *E. coli* strains to swim (Fig. 3A). In contrast, the addition of deferoxamine to swimming plates caused significant changes (Fig. 4A). There was a significant treatment effect of the iron chelator seen in the ability of the bacteria in the outer ring to swim faster, as well in the propensity of the bacteria in the second (or inner ring) to swim slower, in two of the three strains compared to the control. It has been established that there are different nutrients utilized by the bacteria in each of the swimming rings. Those in the first ring consume all of the serine and much of the oxygen, whereas those in the second ring consume aspartic acid (20). The reason that sequestration of iron would cause different swimming rates in the different rings may be a combination of the lack of iron and the depletion of nutrients or oxygen. The gene expression data in this work were based on the bacteria isolated from the outer ring; hence, further investigation into the gene expression changes between swimming rings with or without the addition of iron chelators is warranted.

The addition of FeCl_3_ to swarming plates significantly inhibited swarming (Fig. 3B). This was not unexpected, as iron limitation has been shown to be necessary for swarmer cell differentiation of *Vibrio parahaemolyticus* (21). Addition of the iron chelator to swarming plates had no effect on two of the three strains. The addition of deferoxamine resulted in a significant increase in the swarming area for strain ECC-M. Therefore, the amount of iron available to the bacteria in an unmodified LB swarming plate seems to have been at an optimal level for swarming for two of the strains, whereas a decrease in iron resulting from addition of the chelator resulted in more swarming for one strain (ECC-M).

Because iron can promote swarming and virulence gene expression in bacteria (4), control of iron availability plays an important role in the innate immune system of the host. The host must balance the need for iron, in such essential functions as oxygen transport and energy production through cytochrome *c*, with the need to sequester iron away from pathogens (22, 23). The major iron sequestration protein in the mammary gland is lactoferrin, which is one of the most abundant proteins in the liquid fraction of milk and increases in abundance during infection (24, 25). Neutrophils release lactoferrin with other antimicrobial proteins during a response to pathogens (26). Bacterial growth can be inhibited by the presence of lactoferrin (27). In the case of our three MPEC strains, growth in LB (data not shown), on swimming plates (Fig. 4), and on swarming plates (Fig. 6) was not inhibited by the sequestration or addition of excess iron. In fact, swarming was enhanced in one isolate. An important issue in the understanding of pathogens adapted to the mammary gland would concern their ability to compete for iron in the presence of the host’s iron sequestration mechanisms in milk. Such a mechanism could potentially enable persistent MPEC strains to thrive in an iron-poor environment better than transient mastitis-causing *E. coli* strains.

In addition to the iron acquisition genes, many genes were shown to be differentially expressed in comparisons of growth in LB to growth on swimming or swarming plates. For example, enriched biological process GO terms associated with differentially expressed genes were anaerobic and aerobic respiration, as well as the tricarboxylic acid (TCA) cycle (Table 3). Mutations in genes in the TCA cycle altered swarming patterns of *Proteus mirabilis*, and those bacteria used components of both the aerobic and anaerobic respiratory chains (28). The two genes mutated in that study, *fumC* and *sdhB*, were differentially expressed in our study. The *sdhB* gene was significantly upregulated in both swimming and swarming compared to the level seen in LB, whereas *fumC* was upregulated only in swarming compared to the level in LB.

In conclusion, this report used transcriptomics to compare gene expression changes in three strains of *E. coli* grown under planktonic, swimming, and swarming conditions. Uniquely, we compared results determined under planktonic (growth in liquid media) and swimming (growth on semisolid agar) conditions and demonstrated gene expression differences between these two motility conditions. Our data suggest that swimming motility is an intermediate between planktonic and swarming motility. We also demonstrated the important role that genes associated with iron regulation have in three motility phenotypes. Our research goal is to better understand how pathogens that cause mastitis in dairy cattle establish and maintain an infection and how they evade the host immune response. Our previous research has indicated important differences in motility between *E. coli* strains that cause transient or persistent intramammary infections (8). The motility phenotype important for infection in the mammary gland is unknown. However, since the gland contains a cistern with a large volume of a liquid, all three of these motility types could play a role in infection. When a bacterium enters the gland through the teat sphincter, does it traverse the teat cistern into the main cistern by swimming? Does the bacterium multiply and fill the cistern by planktonic growth? Does it attach and grow along mammary epithelial cells by swarming? Answers to these important questions will lead to a clearer understanding of the mechanisms of pathogenesis in the bovine mammary gland.

## MATERIALS AND METHODS

### Bacterial strains and growth conditions.

The three *E. coli* strains used for these experiments were ECC-Z (O74:H39), ECC-M, (O−:H34) and ECC-1470 (OX18:H−) (7, 29). These strains were isolated from the mammary gland of cows with persistent infections (kind gift from Y. Schukken).

For planktonic conditions, fresh overnight cultures of bacteria were inoculated into fresh liquid Luria-Bertani (LB; 10 g Bacto tryptone–5 g Bacto yeast extract–5 g NaCl–1 liter deionized water) at a 1:1,000 dilution. Bacteria were incubated until mid-log growth (approximate optical density at 600 nm [OD_600_] of 0.7) at 37°C with aeration (200 rpm). For swimming and swarming, a 5-µl aliquot of a fresh liquid LB overnight culture of each of the three *E. coli* strains was plated on 0.3% agar swimming plates (3 g Bacto agar–1 liter LB) or 0.5% agar swarming plates (5 g Bacto agar–0.5% [wt/vol] glucose–1 liter LB) (30, 31). Swimming plates were incubated for approximately 5 h and swarming plates for approximately 12 h in a humid 37°C incubator.

### RNA isolation.

Each of the three MPEC strains was harvested from LB liquid media, swim plates, and swarm plates for RNA isolation. For the LB liquid media, 0.5 ml of each culture was placed in 1.0 ml of RNAProtect (Qiagen, Germantown, MD). For the swim plates, an agar plug was taken from the outermost ring of bacteria using the opposite end of a 200-µl pipette tip, put in 1.0 ml of RNAProtect, and subjected to vortex mixing to disperse it. For the swarm plates, a culture loop was used to take three samples from the outermost edge of the bacteria, with each loopfull being stirred into 1.0 ml of RNAProtect. All samples (9 total [3 MPEC strains under the 3 sets of conditions]) in RNAProtect were processed according to the manufacturer’s instructions. RNA was isolated using an RNeasy minikit (Qiagen, Germantown, MD), followed by genomic DNA removal performed using Turbo DNase DNA-free (Ambion, Austin, TX) according to the product directions. Total RNA quantitation was performed using a NanoDrop ND-1000 spectrophotometer (Thermo Scientific, Wilmington, DE). RNA quality was determined with a 2100 Bioanalyzer (Agilent Technologies, Santa Clara, CA).

### RNA sequencing.

Ribosomal RNA (rRNA) was depleted using a RiboZero rRNA removal kit (bacteria) according to the product instructions (Illumina Inc., San Diego, CA), and rRNA removal was verified on a 2100 Bioanalyzer. Libraries were constructed using TruSeq Stranded Total RNA Library prep kits and were subsequently sequenced on a HiSeq 2500 system using a 100-cycle single-end run (Illumina Inc., San Diego, CA) at the Iowa State University DNA core facility. To ensure robust statistical analyses, we targeted 10 million reads per sample (32); our sequencing resulted in an average of 10.6 ± 0.7 million reads per sample. The quality of the raw reads was assessed using FastQC (33).

### Directional whole-transcriptome RNA sequencing (RNA-Seq) analysis.

CLC Genomics Workbench v7.5 was used to import, filter, and analyze the Illumina sequence data. Sequences from the *E. coli* MG1655 genome (GenBank accession number U00096.3) and an *E. coli* plasmid (GenBank accession number CP009167) from *E. coli* strain 1303, a previous described mastitis-causing strain, were used as reference sequences for mapping the reads using the following parameters: 2 maximum mismatches, 90% minimum length fraction, 80% minimum similarity fraction, and a maximum of 10 hits per read (34). Sense and antisense reads were determined by separately mapping reads to the reverse and forward strands of the reference sequences. Biological replication was achieved by averaging the gene expression data of the three MPEC strains. Gene expression changes were calculated with EdgeR (total count filter cutoff = 5.0) using false-discovery rate (FDR)-corrected *P* values (35) for all pairwise comparisons (LB versus swim; LB versus swarm; swim versus swarm) for each set of data in the sense and antisense directions. The data showing fold changes in gene expression represent the weighted differences between groups based on counts per million calculated in EdgeR. Gene expression differences were considered significant for an FDR *P* of <0.05. A heat map was made using Euclidean distances and complete linkage to compare the expression levels (in normalized log counts per million) from each gene between all the samples.

### Bacterial swimming and swarming assays.

Swimming experiments had 2 technical replicate plates for each strain, and swarming assays had 3 technical replicates. The swimming and swarming experiments were performed 3 times. Each plate was photographed, the width of the diameter of the growth area was measured for swimming assays, and the area of the growth was measured for swarming assays. ImageJ (NIH) was used to determine the diameter and area of the outer bacterial growth circle. The iron chelator deferoxamine or FeCl_3_ (Sigma, St. Louis, MO) or both deferoxamine and FeCl_3_ were added to specific plates at concentrations of 0, 10, 100, and 1,000 µM immediately prior to pouring. Statistical analyses were conducted using Prism version 6 (GraphPad, San Diego, CA). To determine the statistical significance of the results of comparisons between the control and the various dosages of deferoxamine and FeCl_3_, one-way repeated-measure analysis of variance (ANOVA) with Dunnett’s multiple-comparison posttest was used.

### RNAseq data accession number.

The 9 RNAseq raw data files are available on the NCBI website as part of BioProject PRJNA326931.
